# Supplementary material for: The landscape of structural variation in pediatric cancer
Source: bioRxiv. 2025 Apr 26:2025.04.24.650284. Preprint. [Version 1] doi: 10.1101/2025.04.24.650284 (PMC12478399; doi:10.1101/2025.04.24.650284)
Supplement: 1 [file NIHPP2025.04.24.650284V1-supplement-1.pdf]

bioRxiv preprint doi: https://doi.org/10.1101/2025.04.24.650284; this version posted April 26, 2025. The copyright holder for this preprint (which was not certified by peer review) is the author/funder, who has granted bioRxiv a license to display the preprint in perpetuity. It is made available under aCC-BY 4.0 International license.

**Supplementary figures and legends**

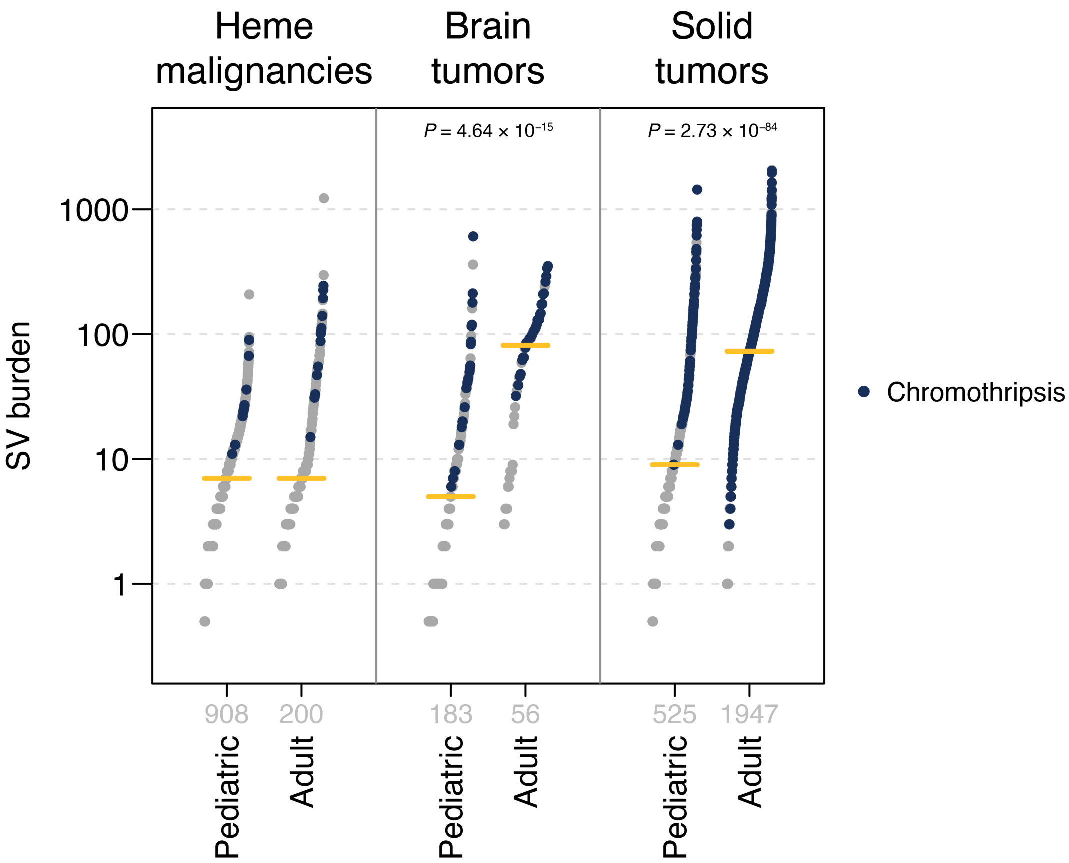


**Supplementary Figure 1. Pediatric and adult SV burden by cancer category.** Plots show the SV burden of pediatric (left) and adult (right) samples in hematological malignancies, brain tumors and solid tumors. Each point represents one sample, blue dots indicate samples with chromothripsis, and median values are indicated with yellow lines. Sample numbers for each cancer type are listed in gray. Significant *P*-values by two-sided Wilcoxon rank-sum tests are listed for categories with significant differences in the SV burden between pediatric and adult samples.

bioRxiv preprint doi: https://doi.org/10.1101/2025.04.24.650284; this version posted April 26, 2025. The copyright holder for this preprint (which was not certified by peer review) is the author/funder, who has granted bioRxiv a license to display the preprint in perpetuity. It is made available under aCC-BY 4.0 International license.

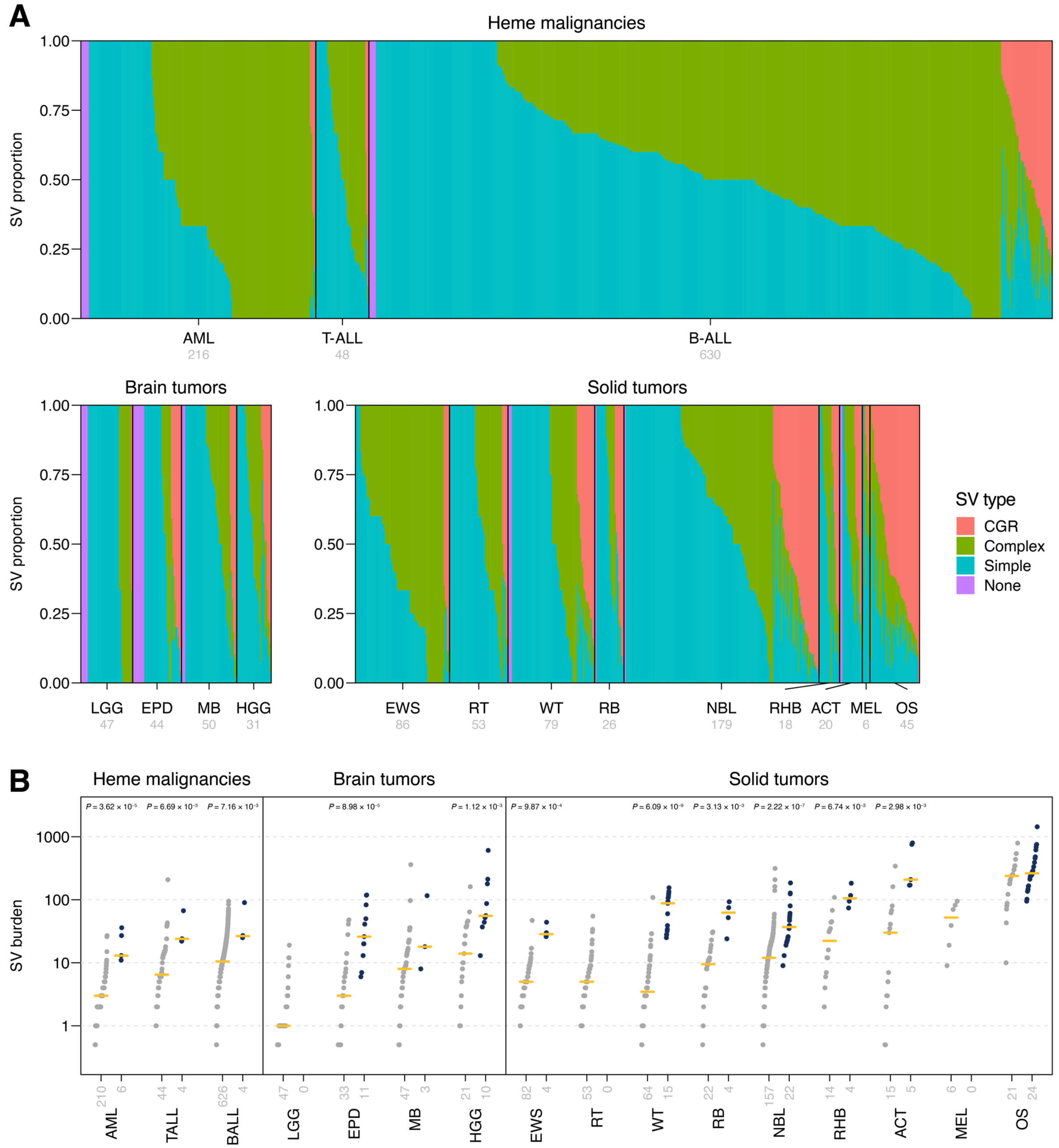


**Supplementary Figure 2. Identification of complex SVs and chromothripsis in pediatric cancers. (a)** Stacked bar chart showing the proportion of structural variants classified as part of complex genome rearrangements (CGR, pink) likely originating from a single event, complex events (green) which refer to additional clustered SVs, or simple events (blue) in each sample. Bars for samples with no detected SVs are shown in magenta. Sample numbers for each cancer type shown in gray. (**b**) SV burden of

bioRxiv preprint doi: https://doi.org/10.1101/2025.04.24.650284; this version posted April 26, 2025. The copyright holder for this preprint (which was not certified by peer review) is the author/funder, who has granted bioRxiv a license to display the preprint in perpetuity. It is made available under aCC-BY 4.0 International license.

chromothripsis-negative (left, gray) and chromothripsis-positive (right, blue) samples, divided by cancer category. Each point represents one sample, and median values are indicated with yellow lines. Sample numbers for each cancer type are listed in gray. Significant *P*-values by two-sided Wilcoxon rank-sum tests are listed for cancers with significant differences in the SV burden between chromothripsis-negative and chromothripsis-positive samples.

bioRxiv preprint doi: https://doi.org/10.1101/2025.04.24.650284; this version posted April 26, 2025. The copyright holder for this preprint (which was not certified by peer review) is the author/funder, who has granted bioRxiv a license to display the preprint in perpetuity. It is made available under aCC-BY 4.0 International license.

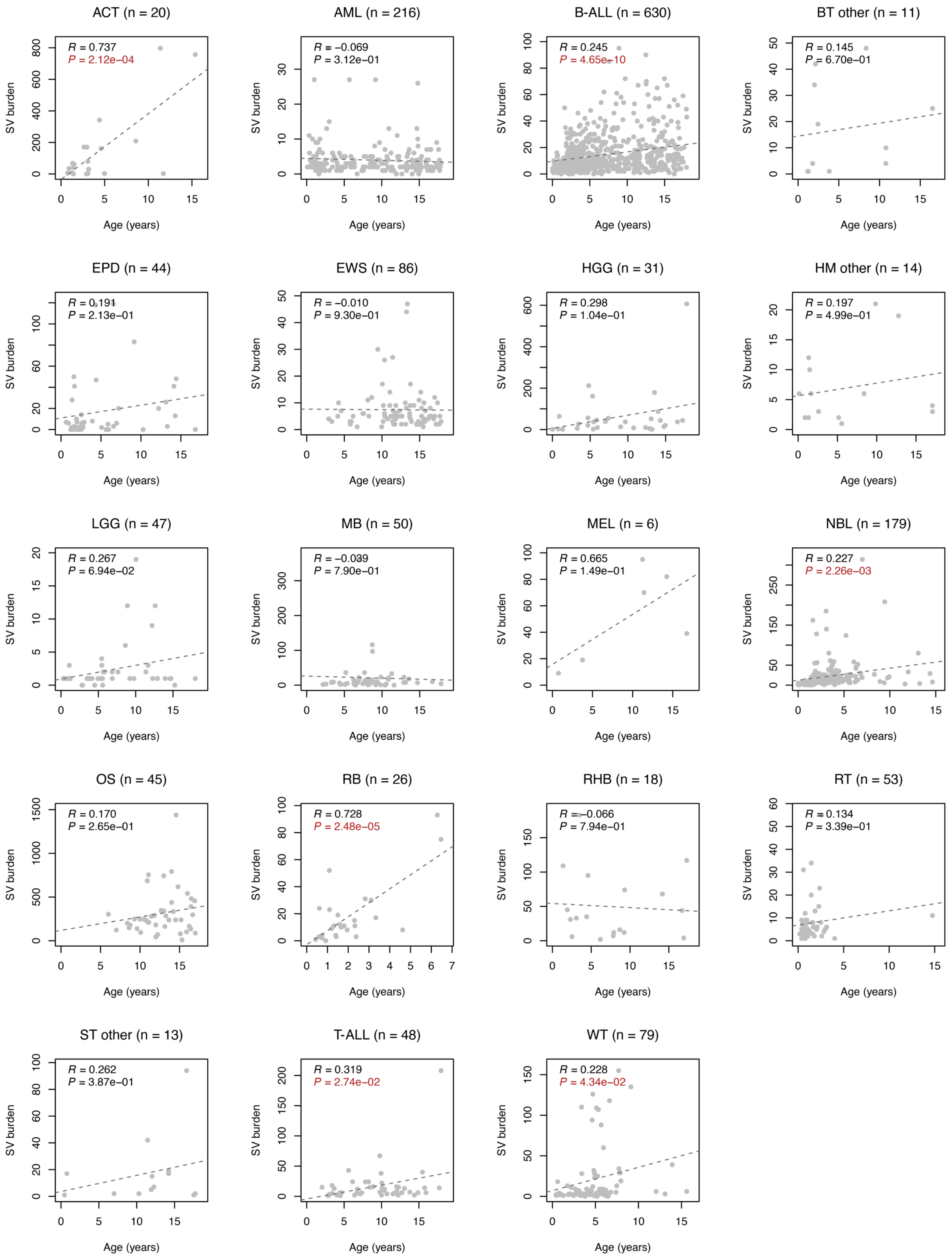

bioRxiv preprint doi: https://doi.org/10.1101/2025.04.24.650284; this version posted April 26, 2025. The copyright holder for this preprint (which was not certified by peer review) is the author/funder, who has granted bioRxiv a license to display the preprint in perpetuity. It is made available under aCC-BY 4.0 International license.

**Supplementary Figure 3. SV burden by age in pediatric cancers.** Each plot shows one pediatric cancer type, with x-axis indicating age at diagnosis and y-axis the total number of SVs in a sample. Each point represents one sample, and sample numbers for each cancer type are shown at top. Pearson *R* and *P*-values are also shown, with significant *P*-values shown in red.

bioRxiv preprint doi: https://doi.org/10.1101/2025.04.24.650284; this version posted April 26, 2025. The copyright holder for this preprint (which was not certified by peer review) is the author/funder, who has granted bioRxiv a license to display the preprint in perpetuity. It is made available under aCC-BY 4.0 International license.

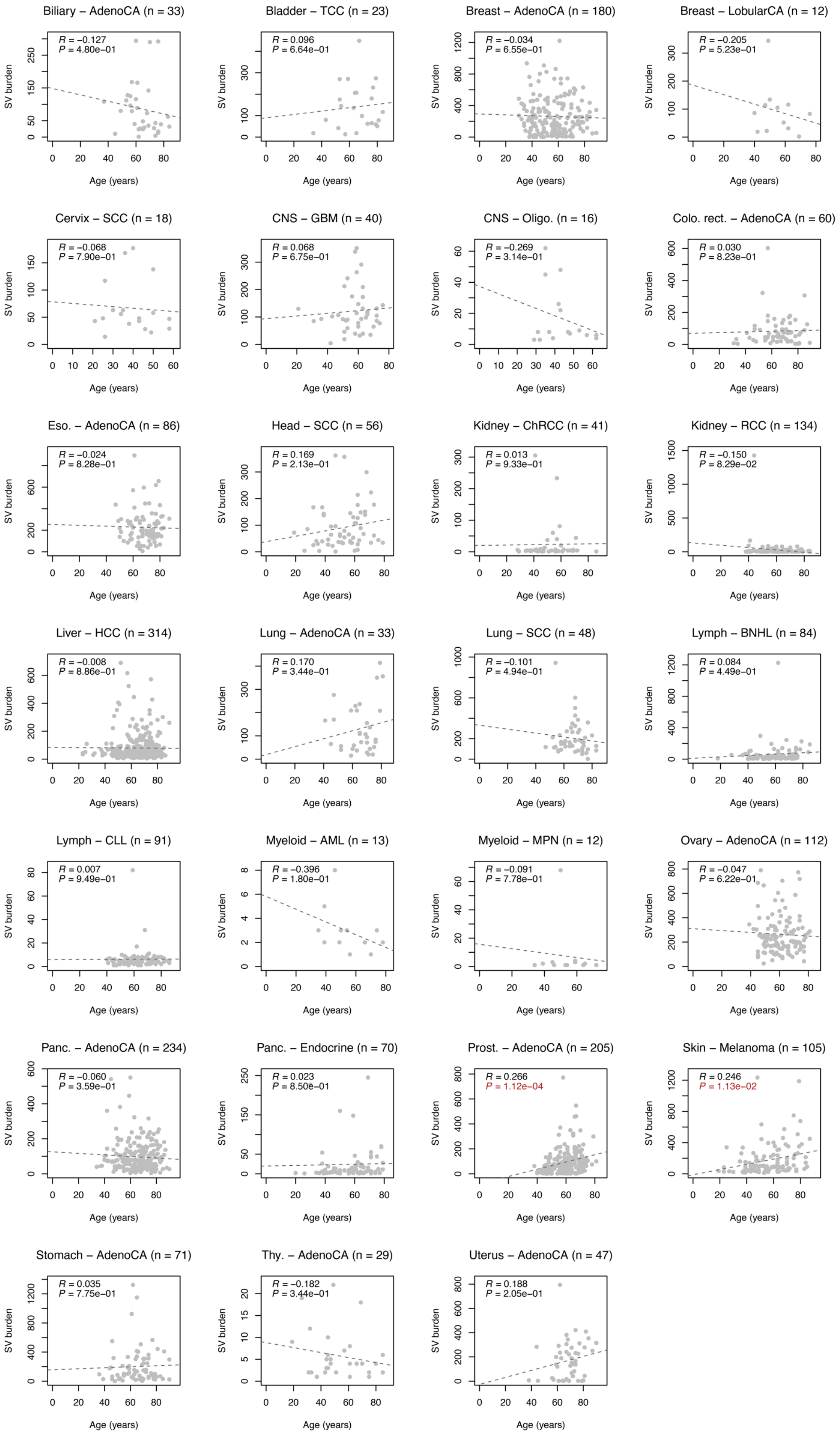

bioRxiv preprint doi: https://doi.org/10.1101/2025.04.24.650284; this version posted April 26, 2025. The copyright holder for this preprint (which was not certified by peer review) is the author/funder, who has granted bioRxiv a license to display the preprint in perpetuity. It is made available under aCC-BY 4.0 International license.

**Supplementary Figure 4. SV burden by age in adult cancers.** Each plot shows one adult cancer type (using PCAWG data), with x-axis indicating age at diagnosis and y-axis the total number of SVs in a sample. Each point represents one sample, and sample numbers for each cancer type are shown at top. Pearson *R* and *P*-values are also shown, with significant *P*-values shown in red.

bioRxiv preprint doi: https://doi.org/10.1101/2025.04.24.650284; this version posted April 26, 2025. The copyright holder for this preprint (which was not certified by peer review) is the author/funder, who has granted bioRxiv a license to display the preprint in perpetuity. It is made available under aCC-BY 4.0 International license.

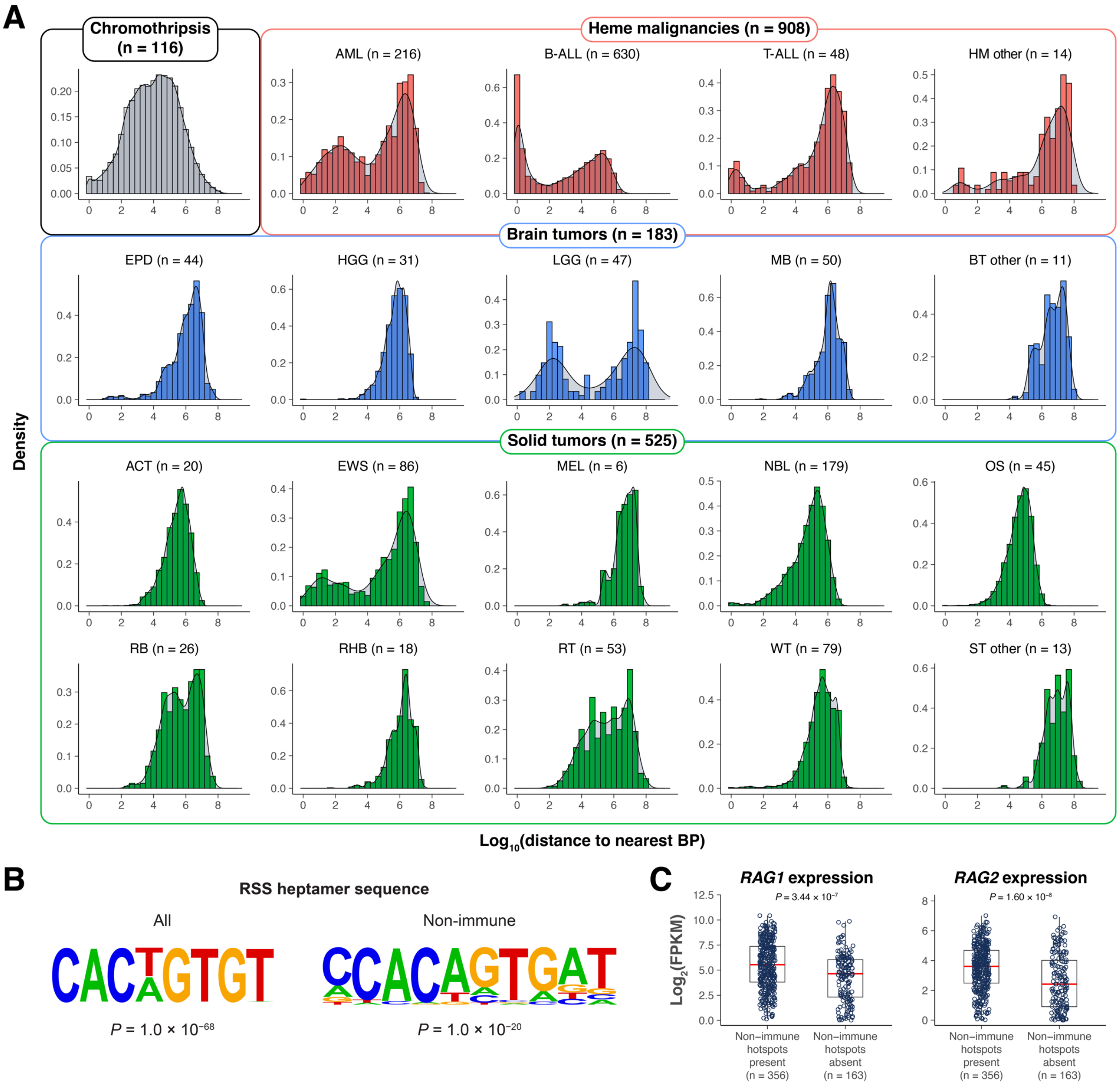


**Supplementary Figure 5. Somatic SV hotspots in pediatric cancer.** (**a**) Distribution of distance to the nearest breakpoint (BP) within chromothripsis-positive samples and in each pediatric cancer type. (**b**) Enrichment of RSS heptamer sequence in all hotspots and non-immune hotspots. (**c**) *RAG1* and *RAG2* expression in B-ALL samples with and without non-immune SV hotspots. *P*-values are obtained via two-sided Wilcoxon rank-sum test.

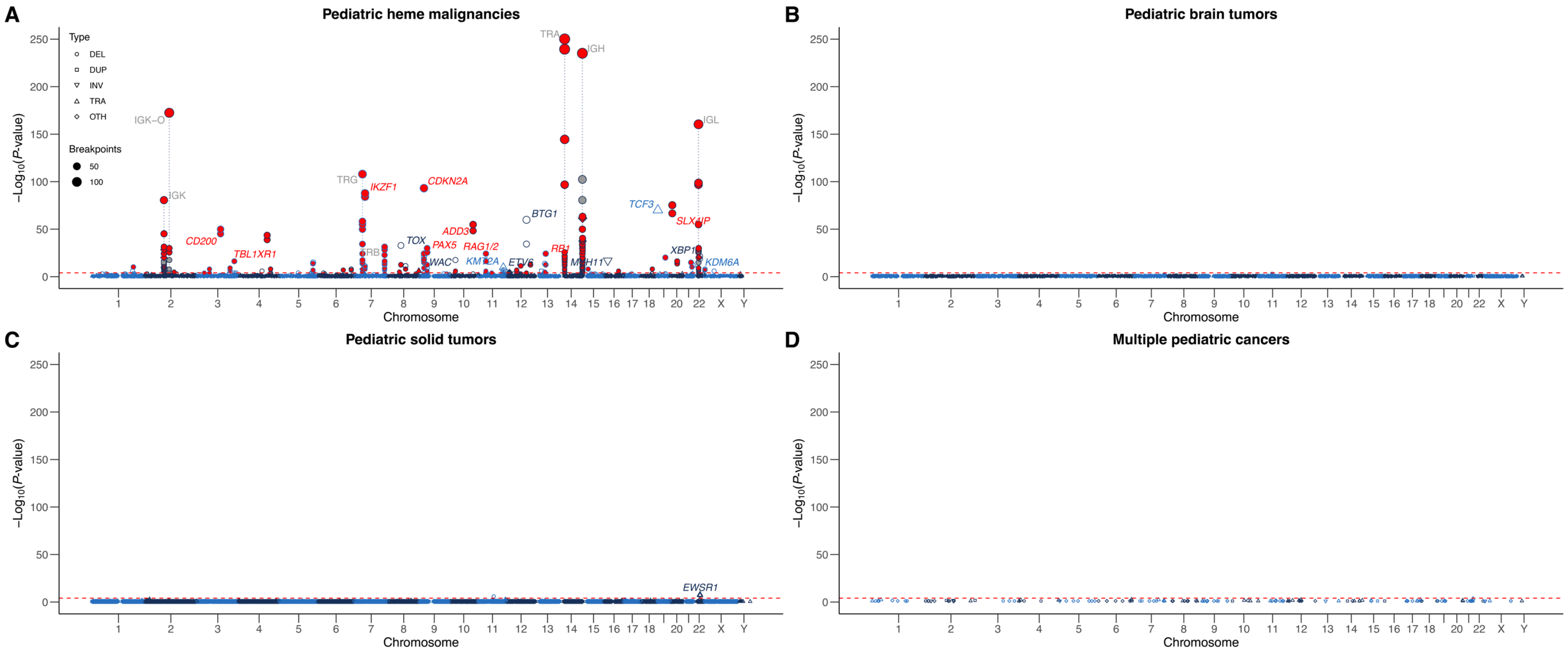


**Supplementary Figure 6. Somatic SV hotspots of pediatric cancer.** Manhattan plots showing SV hotspots contributed by pediatric (**a**) hematological malignancies, (**b**) brain tumors, (**c**) solid tumors, and (**d**) multiple cancer types, and their significance across chromosomes is shown in the same style as Fig. 2D. Hotspots were classified as contributed by heme malignancies, brain tumors, or solid tumors if at least 75% of the breakpoints were contributed by cancers within that category; otherwise, they were assigned to the category of "multiple pediatric cancers".

bioRxiv preprint doi: https://doi.org/10.1101/2025.04.24.650284; this version posted April 26, 2025. The copyright holder for this preprint (which was not certified by peer review) is the author/funder, who has granted bioRxiv a license to display the preprint in perpetuity. It is made available under aCC-BY 4.0 International license.

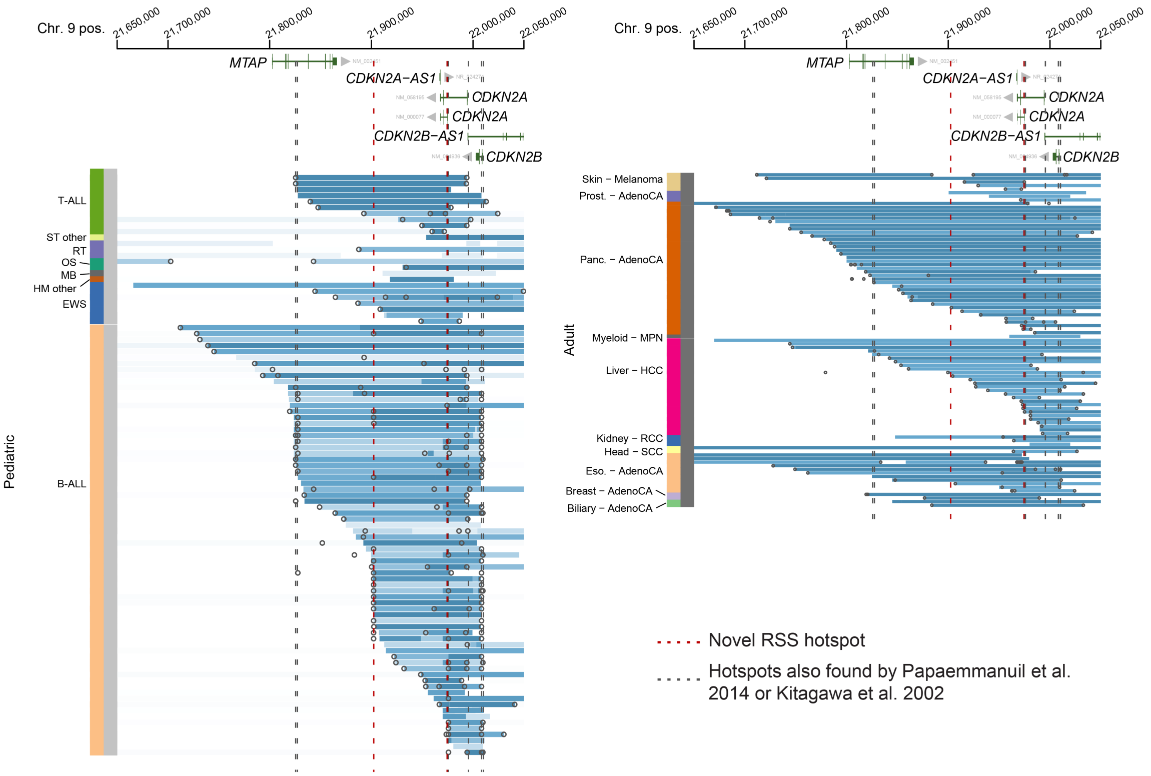


**Supplementary Figure 7. Somatic SV hotspots in *CDKN2A*.** Copy number and SV profiles for pediatric (left) and adult (right) cancers with *CDKN2A* deletions. Each row represents one sample, with cancer type indicated on the left. Circles denote the locations of SV breakpoints while blue coloring indicates copy loss. Vertical dotted lines indicate the locations of RSS associated with SV hotspots in B-ALL found in this study (red color) or reported in previous studies (gray color).

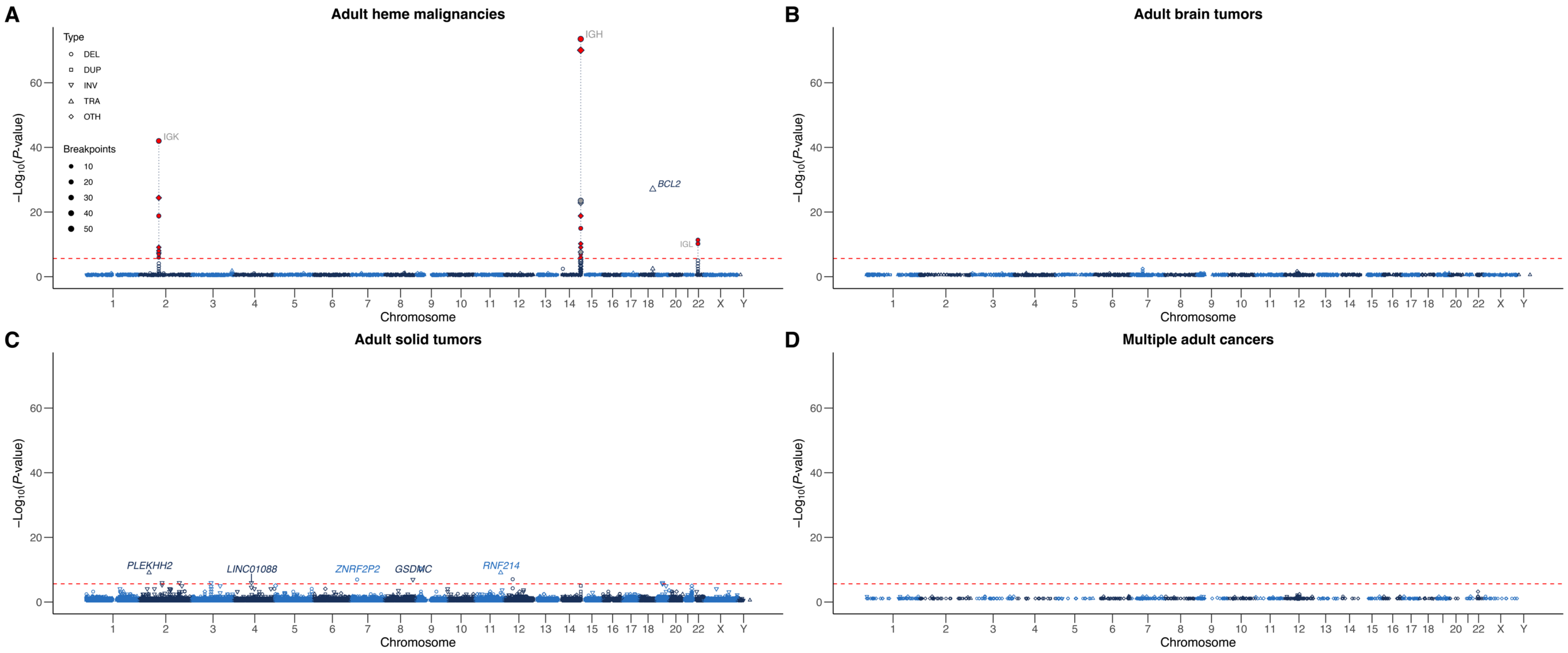


**Supplementary Figure 8. Somatic SV hotspots of adult cancer.** Manhattan plots showing SV hotspots contributed by adult (**a**) heme malignancies, (**b**) brain tumors, (**c**) solid tumors, and (**d**) multiple cancer types, and their significance across chromosomes is presented in the same style as Supplementary Figure 6.

bioRxiv preprint doi: https://doi.org/10.1101/2025.04.24.650284; this version posted April 26, 2025. The copyright holder for this preprint (which was not certified by peer review) is the author/funder, who has granted bioRxiv a license to display the preprint in perpetuity. It is made available under aCC-BY 4.0 International license.

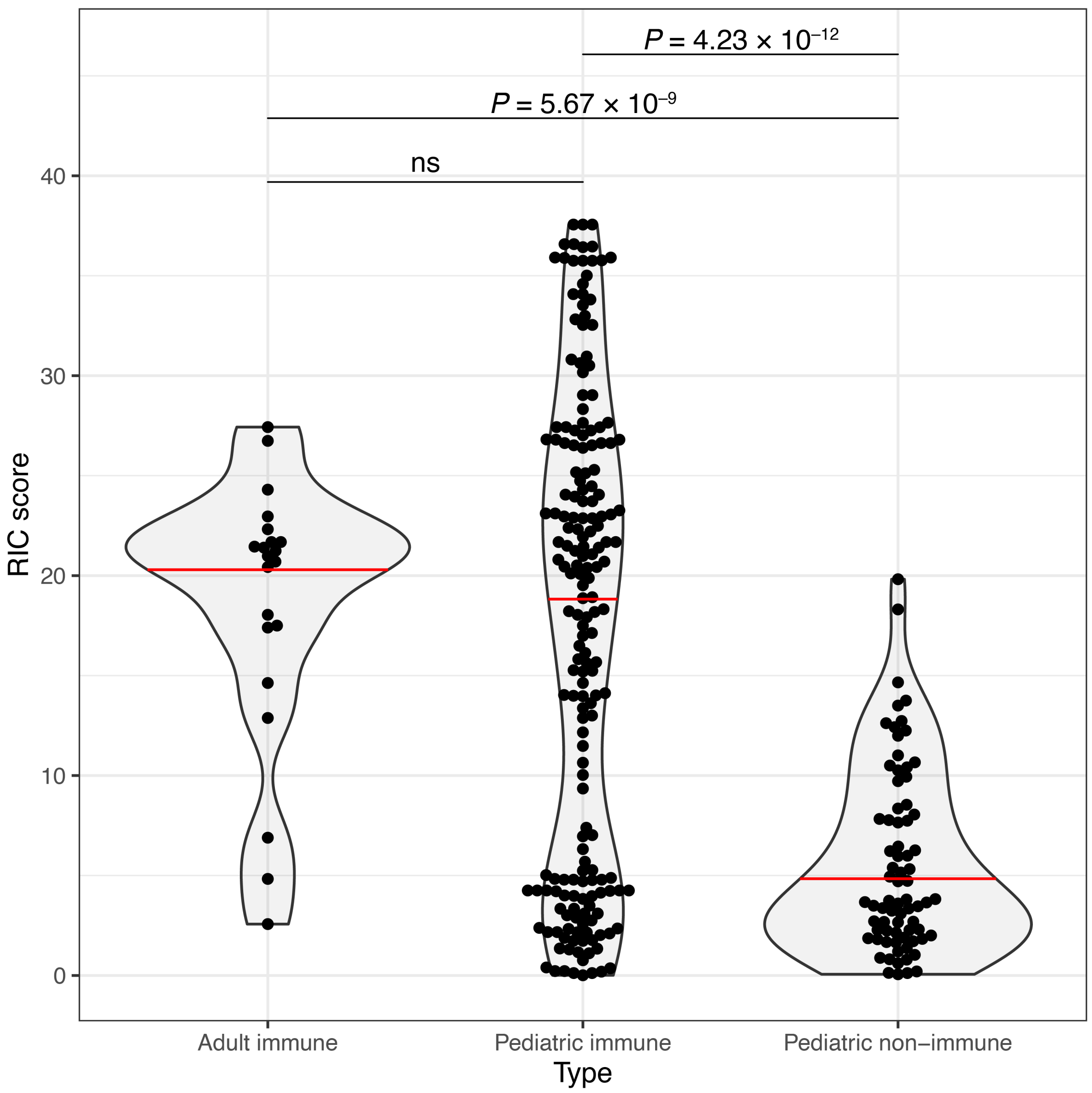


**Supplementary Figure 9. Distribution of recombination information content (RIC) scores of predicted RSS sites in immune and non-immune hotspots in pediatric and adult cancers.** Violin plots of RIC scores associated with hotspots in adult immune regions, and pediatric immune and non-immune regions. For ease of visualization, RIC scores are presented as the score above the threshold value required for positive identification by `RSSsite` (see Methods). Red lines indicate group medians. Significant *P*-values by two-sided Wilcoxon rank-sum tests are listed for each comparison and are corrected for multiple testing using the Benjamini and Hochberg method.

bioRxiv preprint doi: https://doi.org/10.1101/2025.04.24.650284; this version posted April 26, 2025. The copyright holder for this preprint (which was not certified by peer review) is the author/funder, who has granted bioRxiv a license to display the preprint in perpetuity. It is made available under aCC-BY 4.0 International license.

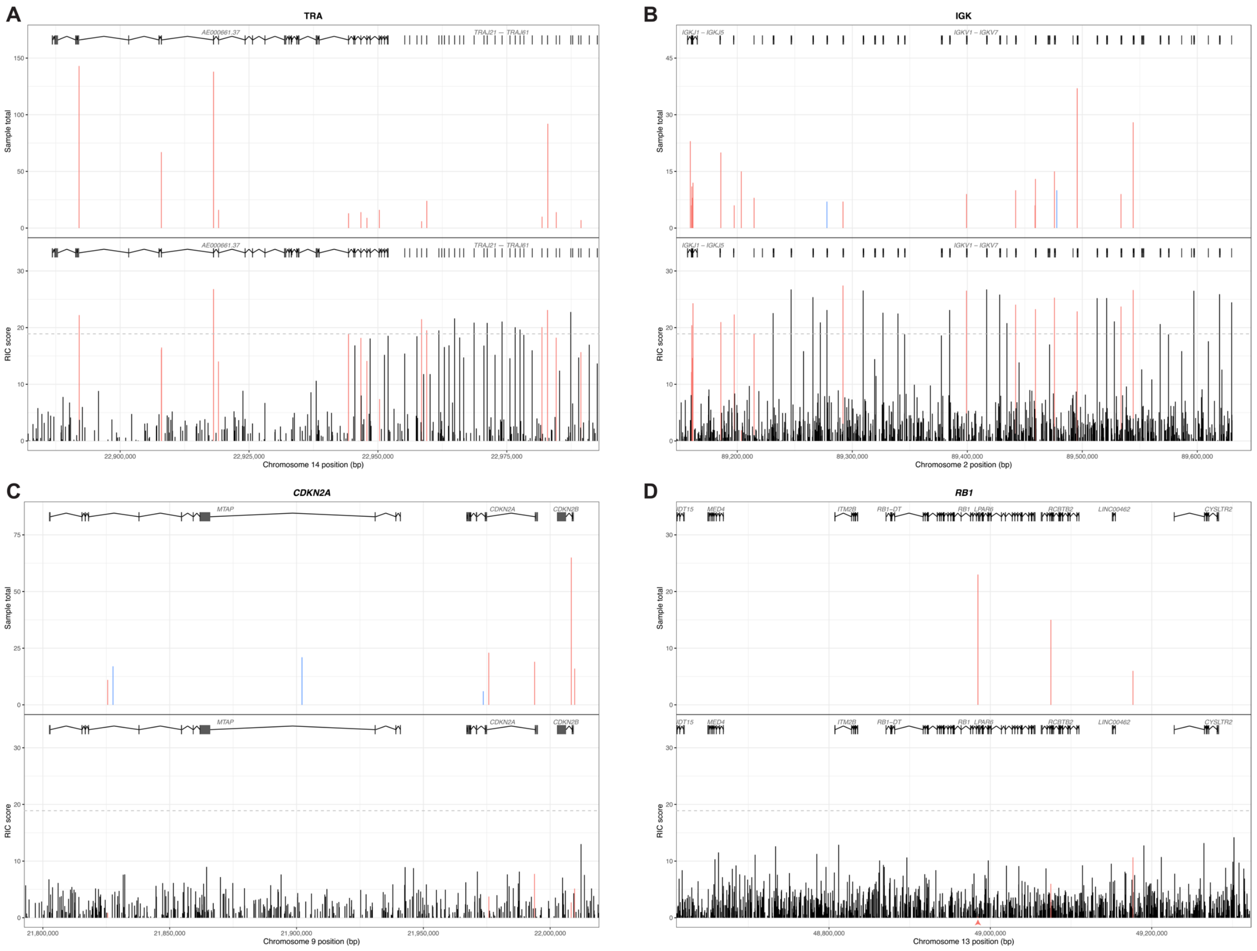


**Supplementary Figure 10. Differential RIC scores of pediatric SV hotspots in representative immune and non-immune regions.** SV hotspots in immune regions are shown in (**a**) TRA and (**b**) IGK while those in non-immune loci are shown in (**c**) *CDKN2A* and (**d**) *RB1*. Gene models are displayed at the top with the x-axis indicating the genomic position. Top panels show the SV hotspots with the total number of samples with SV breakpoints shown on the y-axis and colored by their RSS association status (red: RSS-associated; blue: not RSS-associated). Bottom panels show the distribution of all predicted RSS sites, with the recombination information content (RIC) score given on the y-axis; for ease of visualization, RIC scores are presented as the score above the threshold value required for positive identification by `RSSsite` (see Methods). RSS sites are colored red if associated with a SV hotspot in the top panel. The dotted line indicates the median RIC score for RSS sites associated with pediatric immune region hotspots (18.9). The

bioRxiv preprint doi: https://doi.org/10.1101/2025.04.24.650284; this version posted April 26, 2025. The copyright holder for this preprint (which was not certified by peer review) is the author/funder, who has granted bioRxiv a license to display the preprint in perpetuity. It is made available under aCC-BY 4.0 International license.

red arrowhead in (d) denotes a low-scoring site (0.2 above the minimum threshold RIC score) that is linked to SV breakpoints from 23 samples (i.e. RAG RSS #1 site in Figure 2).

bioRxiv preprint doi: https://doi.org/10.1101/2025.04.24.650284; this version posted April 26, 2025. The copyright holder for this preprint (which was not certified by peer review) is the author/funder, who has granted bioRxiv a license to display the preprint in perpetuity. It is made available under aCC-BY 4.0 International license.

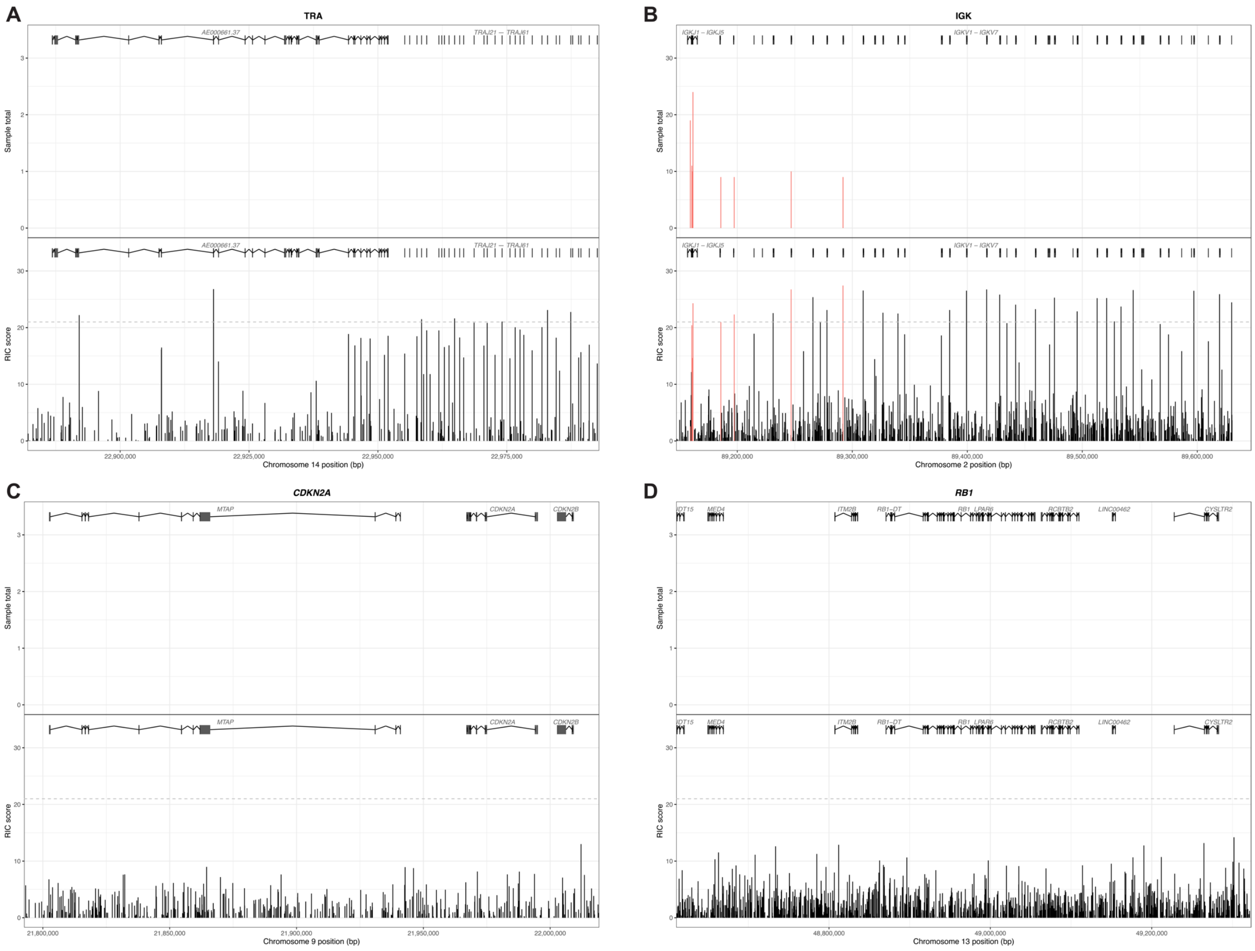


**Supplementary Figure 11. RIC scores of adult SV hotspots in representative immune and non-immune regions.** The SV hotspots were based on adult PCAWG data in the same four regions as those shown for the pediatric SV hotspots in Supplementary Figure 10 using the same style. SV hotspots were detected in IGK (**b**) but absent in TRA (**a**), CDKN2A (**c**), and RB1 (**d**). The dotted line indicates the median RIC score for RSS sites associated with adult immune region hotspots (21.0).

bioRxiv preprint doi: https://doi.org/10.1101/2025.04.24.650284; this version posted April 26, 2025. The copyright holder for this preprint (which was not certified by peer review) is the author/funder, who has granted bioRxiv a license to display the preprint in perpetuity. It is made available under aCC-BY 4.0 International license.

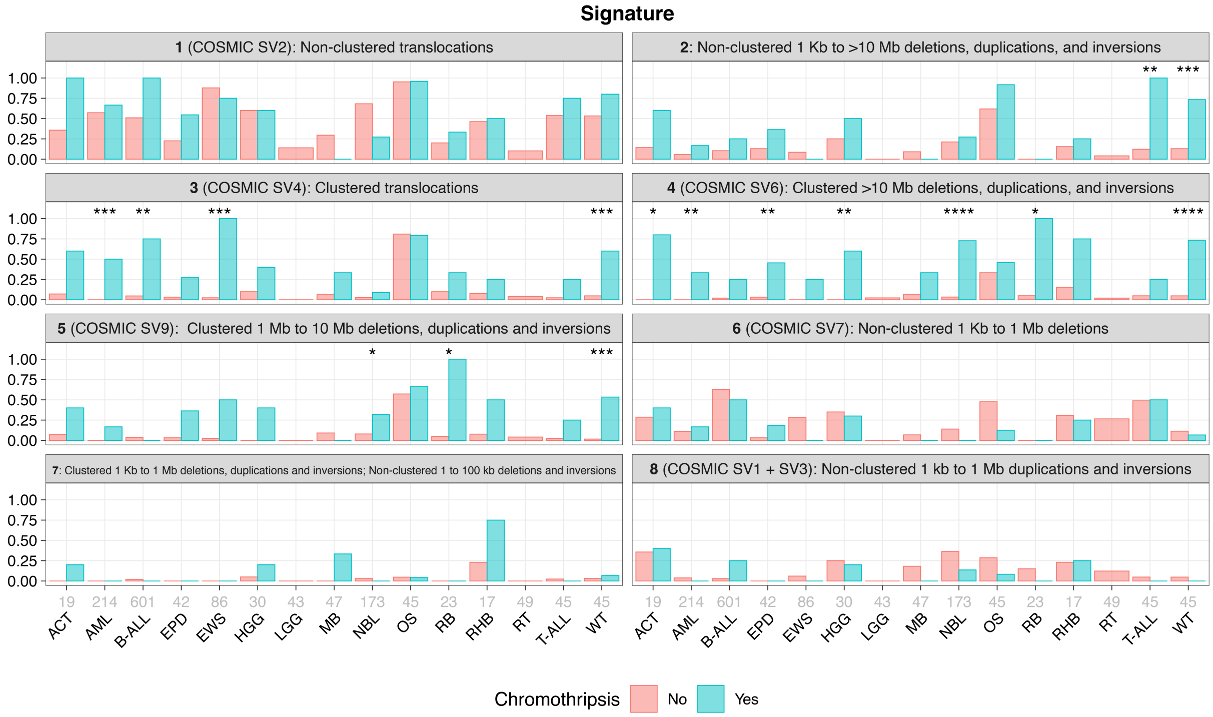


**Supplementary Figure 12. SV signature associations with chromothripsis.** Proportion of chromothripsis-negative (red bar) and chromothripsis-positive samples (blue bar) for all cancer types with at least 15 samples in the pediatric cohort exhibiting each of the eight signatures. Matching COSMIC signatures (if present) and a brief description of each signature are provided. Significant *P*-values by Fisher's exact test are noted for each signature and cancer type with *, **, ***, and ****, which represent *P*-values ≤0.05, 0.01, 0.001, and 0.0001, respectively.

bioRxiv preprint doi: https://doi.org/10.1101/2025.04.24.650284; this version posted April 26, 2025. The copyright holder for this preprint (which was not certified by peer review) is the author/funder, who has granted bioRxiv a license to display the preprint in perpetuity. It is made available under aCC-BY 4.0 International license.

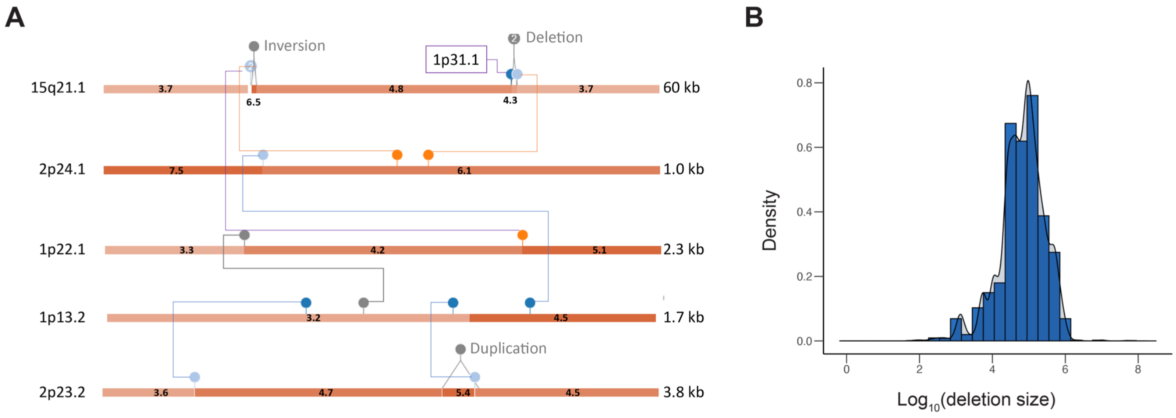


**Supplementary Figure S13. Illustration of features representative of pediatric SV signatures 3 and 6.** (**a**) An example of clustered inter-chromosomal events in SJOS013, the defining feature of pediatric SV signature 3. This event involves five distinct regions of chromosomes 1, 2, and 15 contain 2–4 rearrangements within 1–2 kb. SVs defined as inter-chromosomal translocations are shown with colored dots while intra-chromosomal events are shown with gray dots. (**b**) Histogram of deletion sizes associated with RSS hotspots. The deletion sizes match the non-clustered deletions comprising pediatric SV signature 6 (i.e. non-clustered deletion between 1 Kb – 1 Mb), which is enriched in pediatric B-ALL and T-ALL.

bioRxiv preprint doi: https://doi.org/10.1101/2025.04.24.650284; this version posted April 26, 2025. The copyright holder for this preprint (which was not certified by peer review) is the author/funder, who has granted bioRxiv a license to display the preprint in perpetuity. It is made available under aCC-BY 4.0 International license.

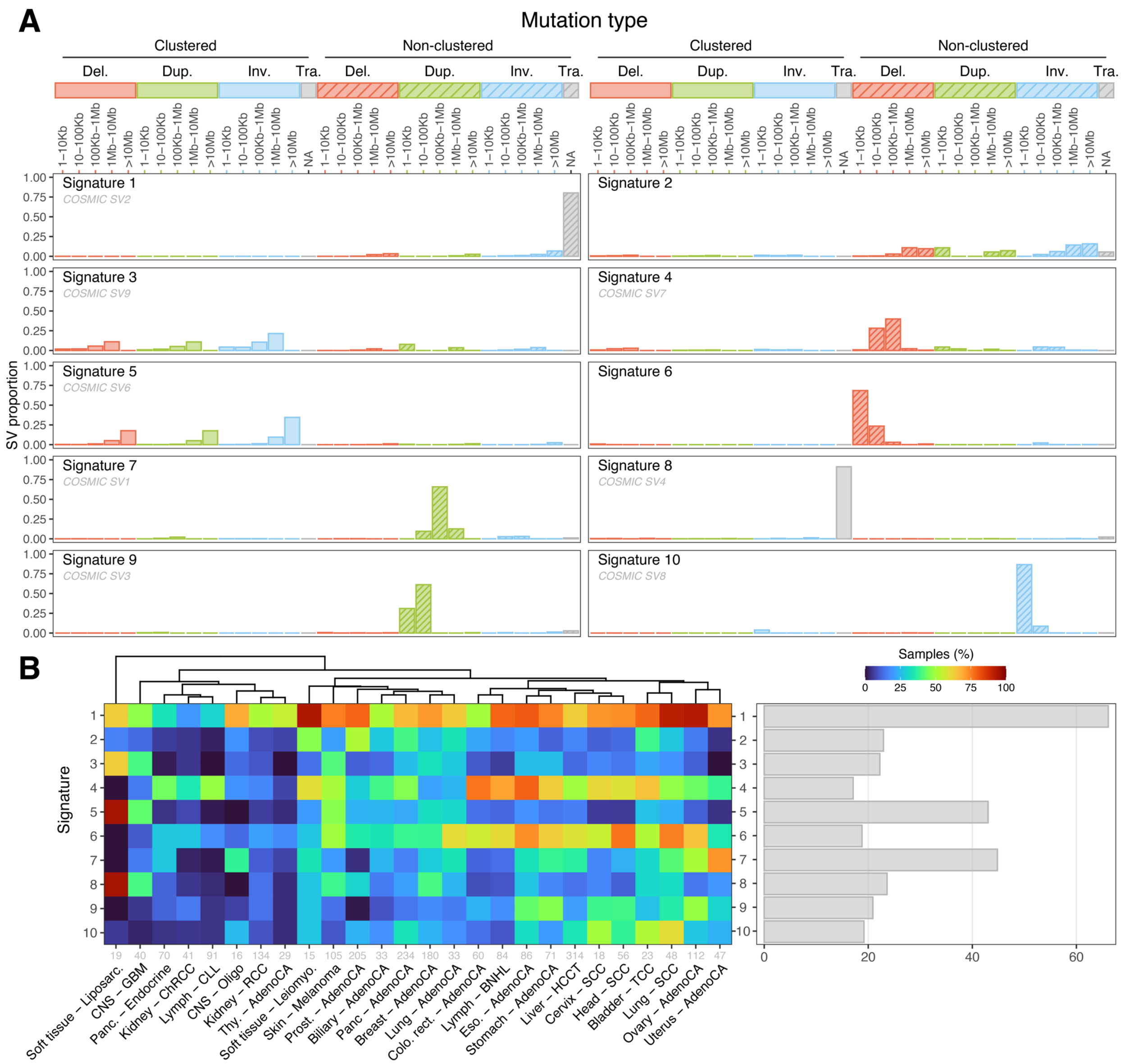

**Figure 14. Somatic SV signatures in adult cancer.** (**a**) Bar plots showing the distributions of the 32 SV types in all ten SV signatures extracted from the adult cancer cohort. Non-clustered signatures are represented by patterned bars. Signatures with matches to the COSMIC database have their corresponding COSMIC signature labeled in gray. (**b**) Heatmap showing the percent of cancer samples with each of the SV signatures shown in (a) for all cancer types with at least 15 samples. The bar plot at right shows the percentage of all adult cancer samples exhibiting each signature.

bioRxiv preprint doi: https://doi.org/10.1101/2025.04.24.650284; this version posted April 26, 2025. The copyright holder for this preprint (which was not certified by peer review) is the author/funder, who has granted bioRxiv a license to display the preprint in perpetuity. It is made available under aCC-BY 4.0 International license.

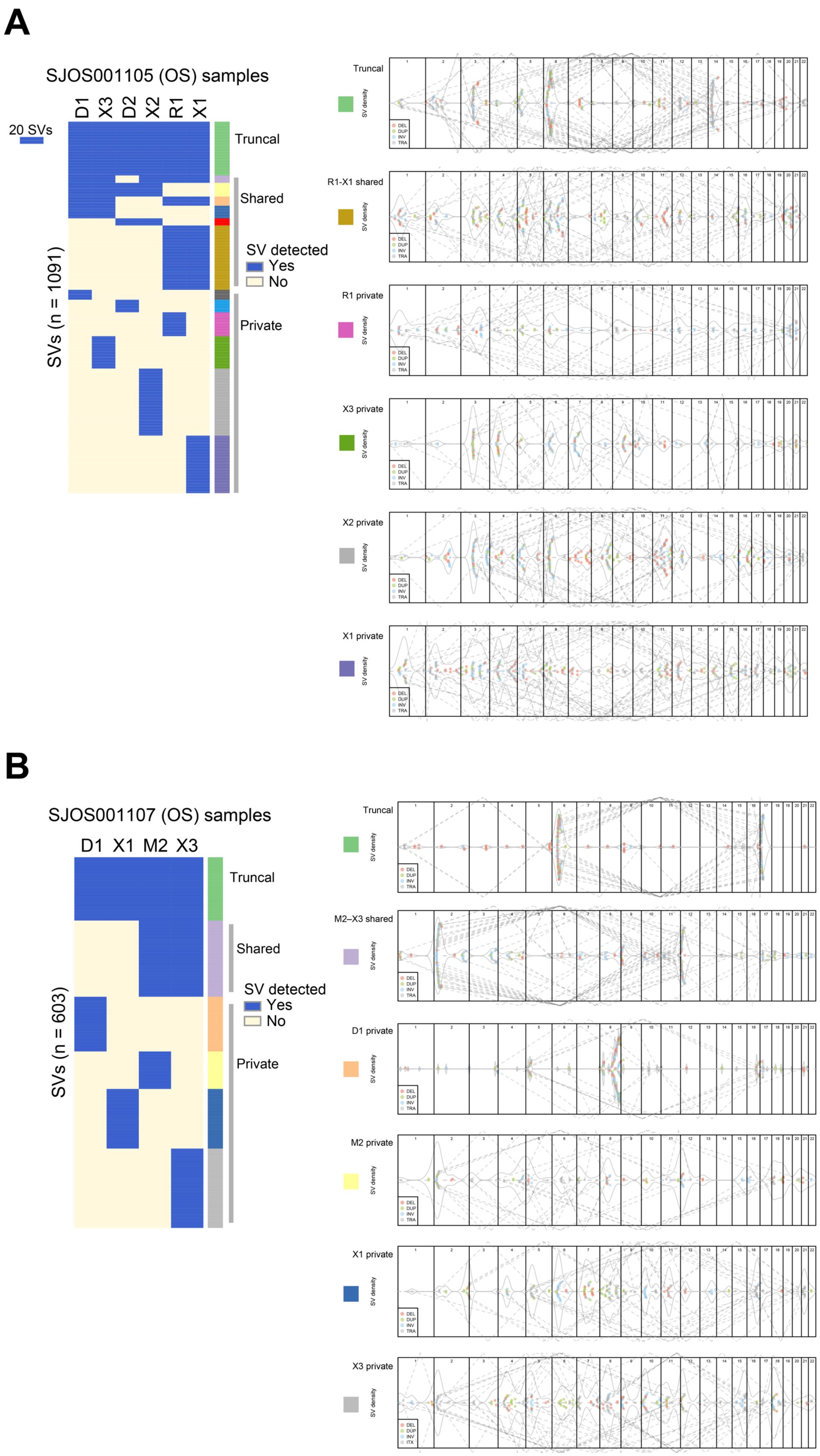

bioRxiv preprint doi: https://doi.org/10.1101/2025.04.24.650284; this version posted April 26, 2025. The copyright holder for this preprint (which was not certified by peer review) is the author/funder, who has granted bioRxiv a license to display the preprint in perpetuity. It is made available under aCC-BY 4.0 International license.

**Supplementary Figure 15. SV temporal evolution in two multi-sample osteosarcoma patients.** SV grouping (left) and genome-wide density plot (right) shown in the same style as Figure 4. (**a**) Diagnostic (D1) and metastatic (D2 and R1) tumors profiled for patient SJOS001105 with D2 and R1 samples acquired two years after D1. X3, X1 and X2 are from PDX models developed from D1, R1 and M2, respectively. (**b**) Diagnostic (D1) and metastatic (M2) tumors profiled for patient SJOS001107, with the M2 sample acquired one year after D1. X1 and X3 are from PDX models developed from D1 and M2, respectively. In both patients, presence of SVs private to PDX samples indicates that PDX samples appear to grow out of one of the clones of the primary patient tumor samples and may have continued SV evolution.

bioRxiv preprint doi: https://doi.org/10.1101/2025.04.24.650284; this version posted April 26, 2025. The copyright holder for this preprint (which was not certified by peer review) is the author/funder, who has granted bioRxiv a license to display the preprint in perpetuity. It is made available under aCC-BY 4.0 International license.

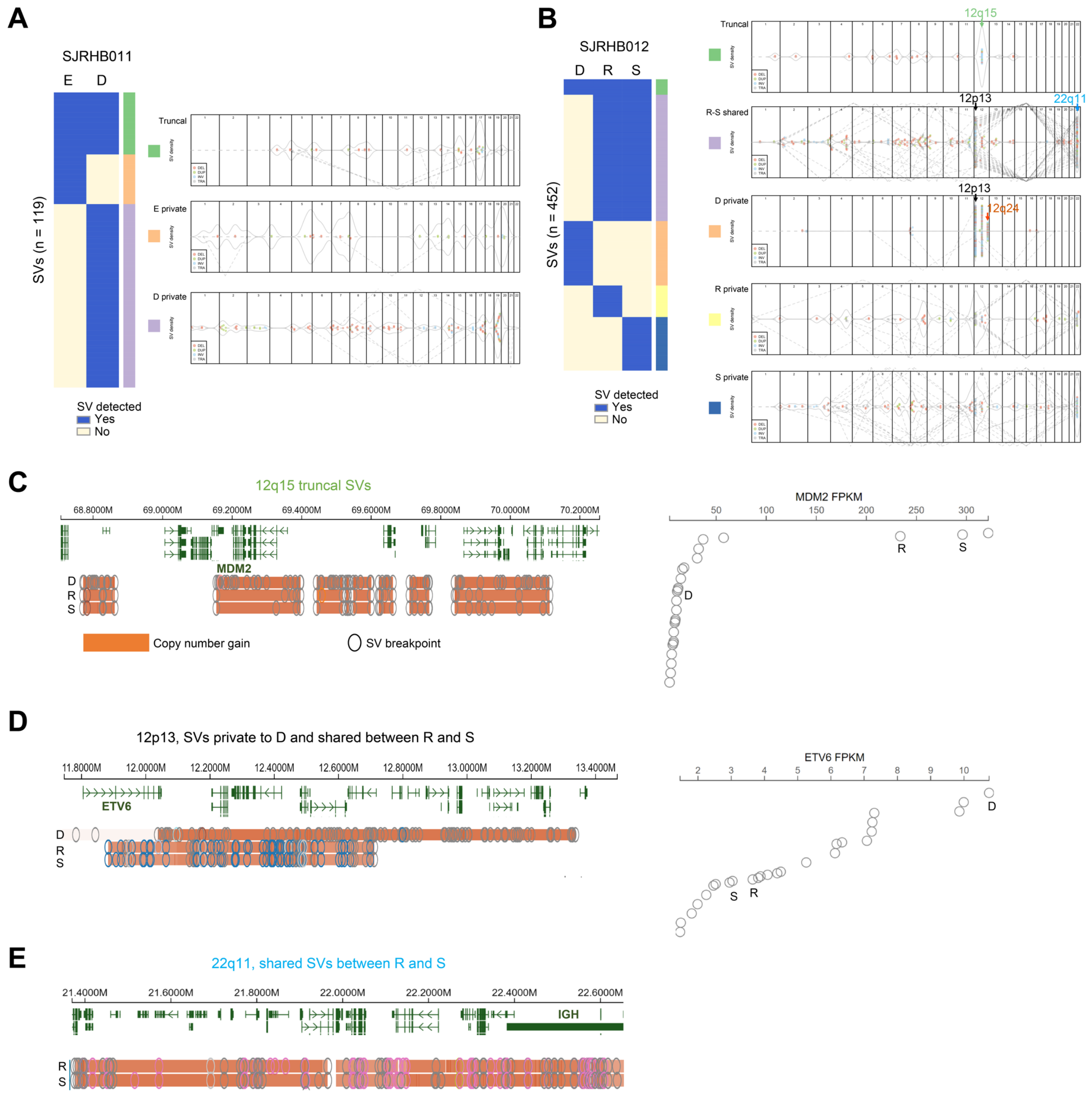


**Supplementary Figure 16. SV evolution in two embryonal rhabdomyosarcoma (ERMS) patients. (a)** SV grouping (left) and genome-wide density plot (right) shown in the same style as Figure 4 for two RHB samples obtained from one patient. In this particular case, E refers to tumor sample acquired at diagnosis and D refers to a relapsed tumor acquired 15 months later. (**b**) As in (a), except that a different patient is shown. In this case, D was from diagnosis at prostate while R and S were from relapsed tumors at prostate and pelvis acquired 14 months later. Chromosome 12 had three different groups of SVs: 12q15 with truncal variants (shown in **c**), 12p13 which can form either a shared SV group joining 22q11 (details in **d** and **e**) or

bioRxiv preprint doi: https://doi.org/10.1101/2025.04.24.650284; this version posted April 26, 2025. The copyright holder for this preprint (which was not certified by peer review) is the author/funder, who has granted bioRxiv a license to display the preprint in perpetuity. It is made available under aCC-BY 4.0 International license.

a private SV group joining 12q23. (**c**) Truncal copy-number gain (left) leading to increased *MDM2* expression (right), particularly in R and S in sample SJRB012 shown in (b). The gene expression data were based on all ERMS samples hosted on the GenomePaint portal (https://viz.stjude.cloud/tools/genomepaint). (**d**) Private SVs leading to increased *ETV6* expression in D; shared SVs impacting R and S are present in the same region. (**e**) Shared SVs between R and S impacting the *IGH* locus.

bioRxiv preprint doi: https://doi.org/10.1101/2025.04.24.650284; this version posted April 26, 2025. The copyright holder for this preprint (which was not certified by peer review) is the author/funder, who has granted bioRxiv a license to display the preprint in perpetuity. It is made available under aCC-BY 4.0 International license.

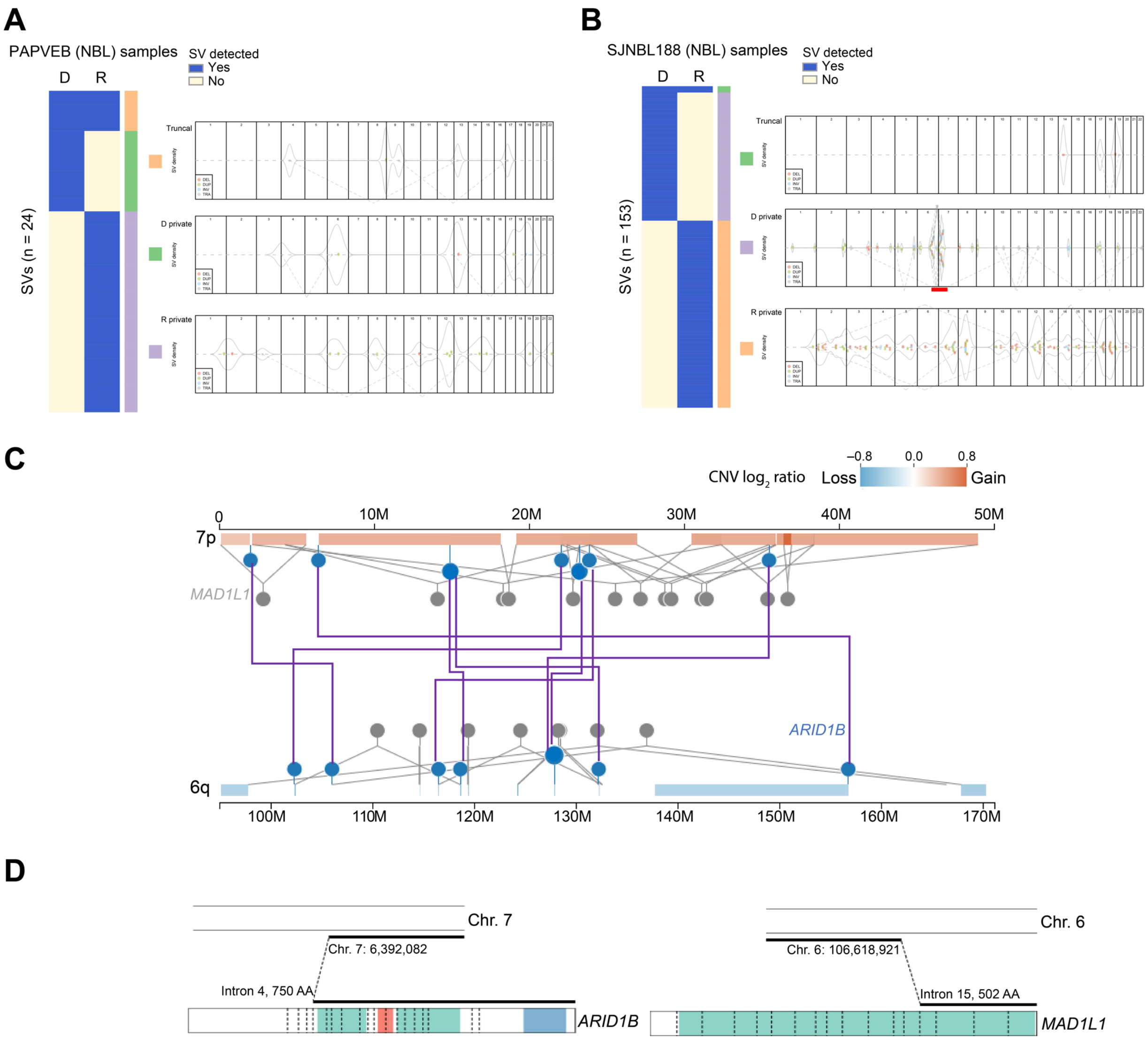


**Supplementary Figure 17. SV evolution in two multi-sample neuroblastoma patients.** (**a**) SV grouping (left) and genome-wide density plot (right) shown in the same style as Figure 4 for two NBL samples obtained from one patient. D is from diagnosis while R is a relapse. (**b**) As in (a), except that a different patient is shown. D is from diagnosis while R is a relapse. The red bar at the 6q–7p region on the beeswarm plot for the SV group “D private” indicates a chromothripsis event. (**c**) Integrated view of SVs and CNVs at the 6q–7p region in (b) (region highlighted by a red bar) associated with a chromothripsis event targeting the *ARID1B* neuroblastoma driver gene. (**d**) Schematic detailing how this event disrupted both *ARID1B* and the common fragile site gene *MAD1L1*.

bioRxiv preprint doi: https://doi.org/10.1101/2025.04.24.650284; this version posted April 26, 2025. The copyright holder for this preprint (which was not certified by peer review) is the author/funder, who has granted bioRxiv a license to display the preprint in perpetuity. It is made available under aCC-BY 4.0 International license.

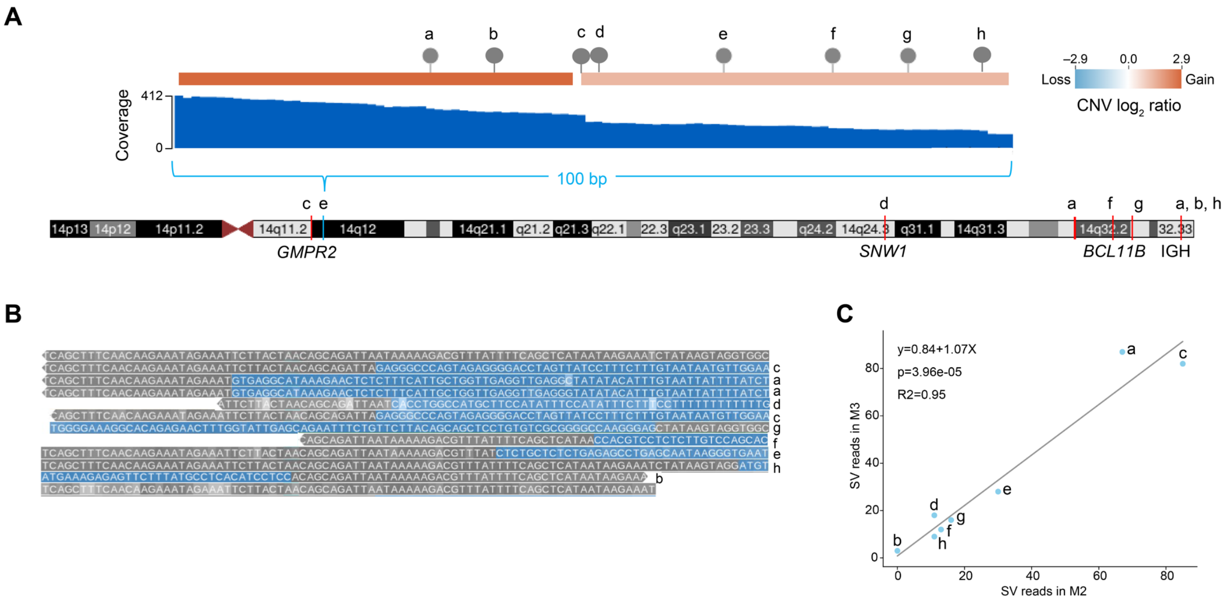


**Supplementary Figure 18. An amplicon with a high-density SV cluster defined as "shared" in multi-region analysis of osteosarcoma patient SJOS00101.** This is a detailed view for the region shown in Figure 4b. (**a**) Eight SVs within 100 bp in an amplicon connected to seven distinct regions of chromosome 14. (**b**) Reference (top) and SV junction sequence (blue) at each of the eight breakpoints matching the labels in (a). (**c**) Concordant SV junction read counts between M2 and M3 samples. This suggests these may all be linked to a single, late event.

bioRxiv preprint doi: https://doi.org/10.1101/2025.04.24.650284; this version posted April 26, 2025. The copyright holder for this preprint (which was not certified by peer review) is the author/funder, who has granted bioRxiv a license to display the preprint in perpetuity. It is made available under aCC-BY 4.0 International license.

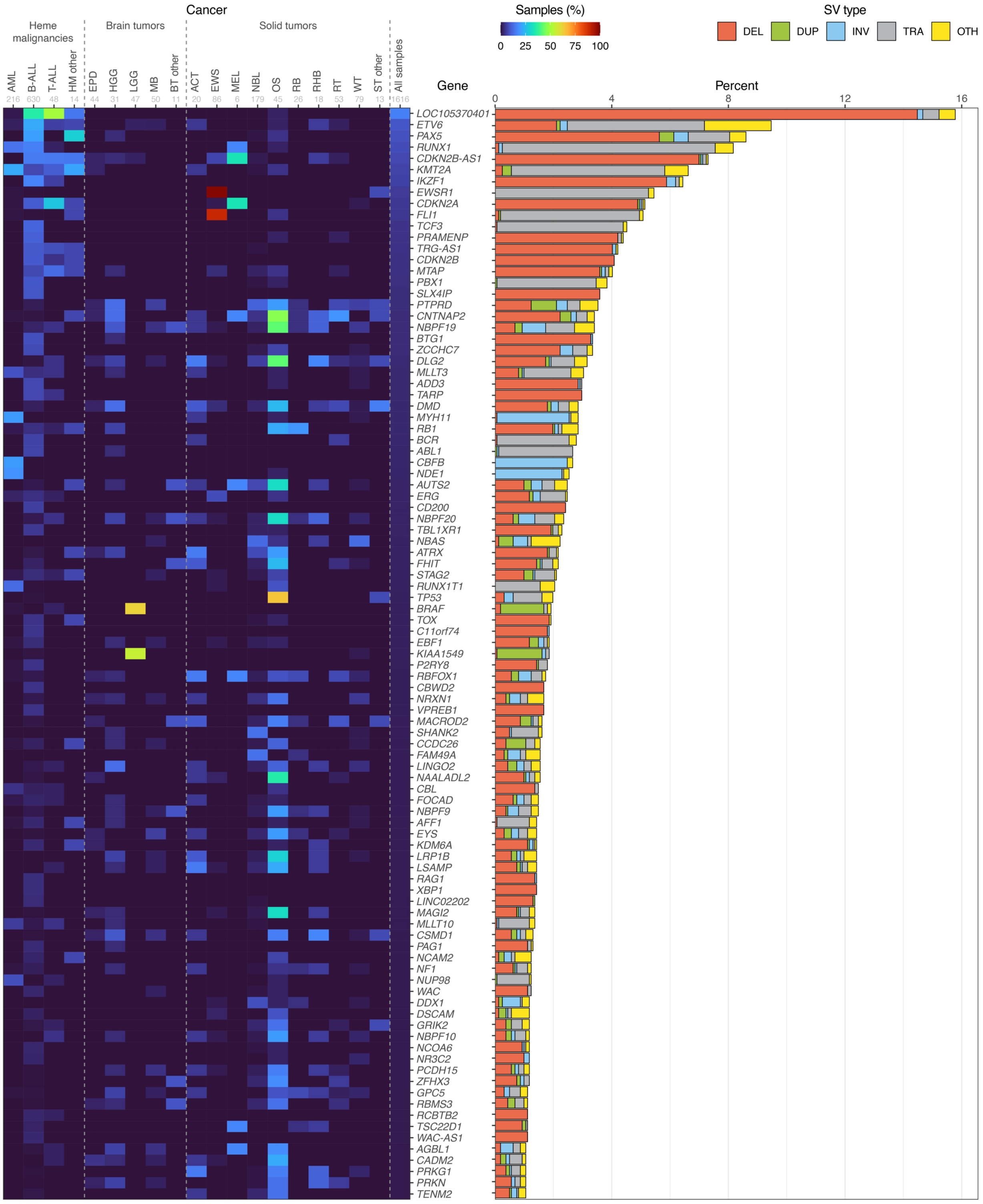


**Supplementary Figure 19. Frequency of intragenic SVs.** Heatmap showing the percentage of samples in each pediatric cancer type (column) with somatic SVs impacting each gene (row). A gene was considered impacted in a sample if an SV breakpoint fell within any part of the gene including both exonic and intronic

bioRxiv preprint doi: https://doi.org/10.1101/2025.04.24.650284; this version posted April 26, 2025. The copyright holder for this preprint (which was not certified by peer review) is the author/funder, who has granted bioRxiv a license to display the preprint in perpetuity. It is made available under aCC-BY 4.0 International license.

regions. Genes with SVs in at least 1% of pediatric cancer samples are shown and their order is sorted based on the prevalence of the entire cohort. Bar plot at right shows the percent of all pediatric cancer samples with SVs in each gene, separated by SV type. When multiple SV types within the same sample impacted the same gene, they are denoted with OTH.

bioRxiv preprint doi: https://doi.org/10.1101/2025.04.24.650284; this version posted April 26, 2025. The copyright holder for this preprint (which was not certified by peer review) is the author/funder, who has granted bioRxiv a license to display the preprint in perpetuity. It is made available under aCC-BY 4.0 International license.

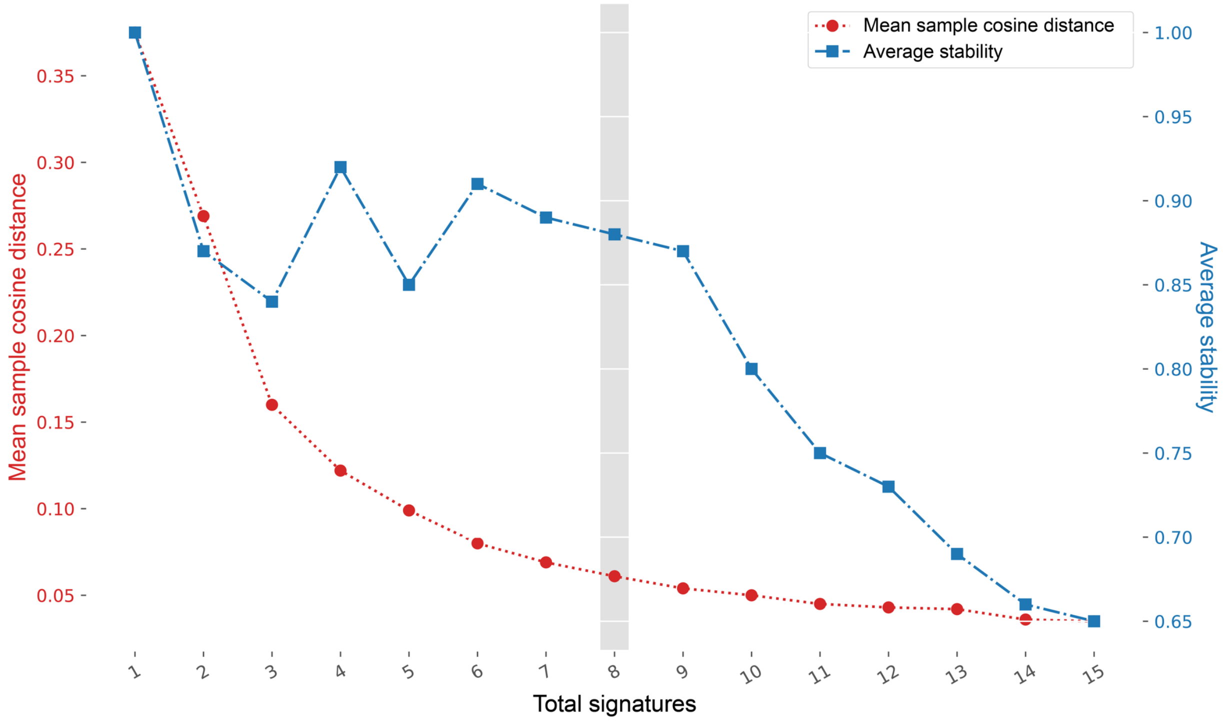


**Supplementary Figure 20. `SigProfiler` optimal selection plot for the pediatric cohort.** Optimal number of signatures for the pediatric cohort as determined by `SigProfiler`. Left y-axis (red): Mean sample cosine distance; right y-axis (blue): average stability; x-axis: total number of signatures.

bioRxiv preprint doi: https://doi.org/10.1101/2025.04.24.650284; this version posted April 26, 2025. The copyright holder for this preprint (which was not certified by peer review) is the author/funder, who has granted bioRxiv a license to display the preprint in perpetuity. It is made available under aCC-BY 4.0 International license.

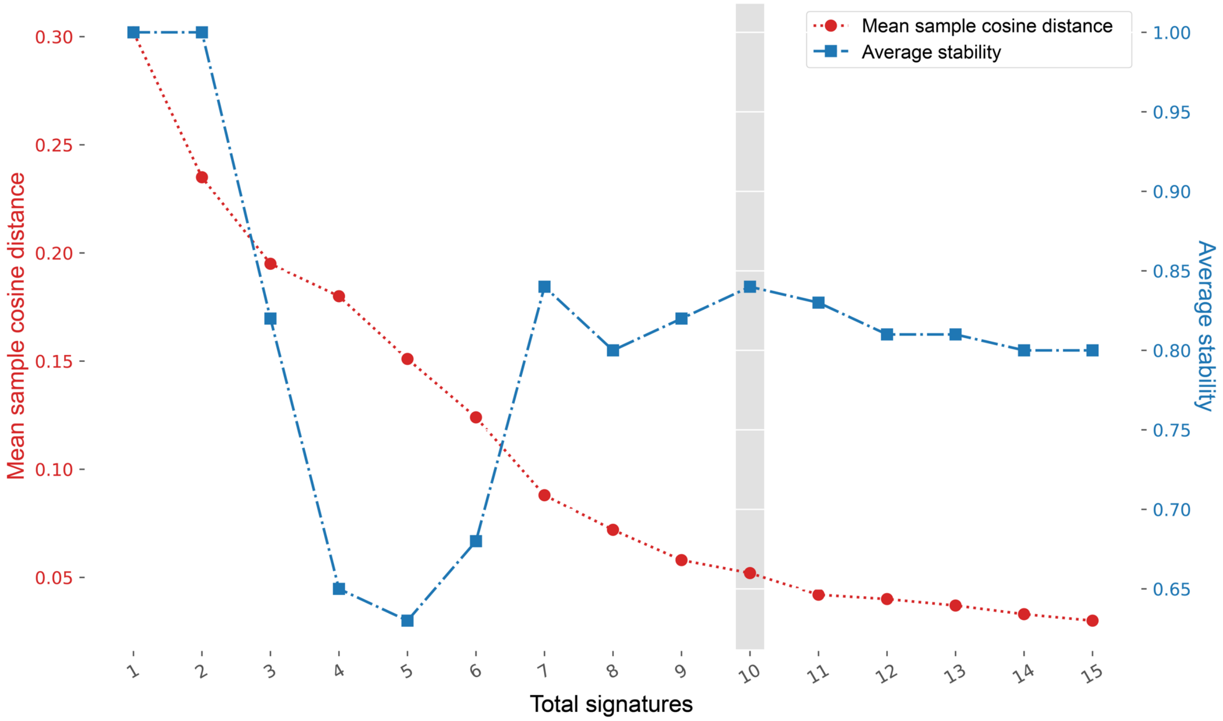


**Supplementary Figure 21. `SigProfiler` optimal selection plot for the adult cohort.** Optimal number of signatures for the adult cohort as determined by `SigProfiler`. Left y-axis (red): Mean sample cosine distance; right y-axis (blue): average stability; x-axis: total number of signatures.
